# Effectiveness of a Structured Disaster Management Training Program on Nurses' Disaster Readiness for Response to Emergencies and Disasters: A Randomized Controlled Trial

**DOI:** 10.1155/2024/5551894

**Published:** 2024-07-08

**Authors:** Chia-Huei Lin, Wen-Chii Tzeng, Li-Chi Chiang, Min-Chin Lu, Meei-Shyuan Lee, Shang-Lin Chiang

**Affiliations:** ^1^ School of Nursing & Graduated Institute of Medical Sciences National Defense Medical Centre, Taipei, Taiwan; ^2^ Department of Nursing Tri-Service General Hospital, Taipei, Taiwan; ^3^ School of Public Health National Defense Medical Centre, Taipei, Taiwan; ^4^ Department of Physical Medicine and Rehabilitation Tri-Service General Hospital School of Medicine National Defense Medical Center, Taipei, Taiwan

## Abstract

**Background:**

Most frontline nurses lack sufficient readiness for effective disaster response. Therefore, designing a disaster management training program (DMTP) to promote nurses' readiness for disaster response is imperative.

**Aim:**

This study aimed to evaluate the effectiveness of a structured DMTP on nurses' readiness for response to disasters.

**Methods:**

A randomized controlled trial was conducted. One hundred eligible nurses, recruited using convenience sampling from a medical centre in northern Taiwan, were randomly assigned to either the experimental (EG, *n* = 50) or control (CG, *n* = 50) group. Both groups received regular continuous nursing education. The EG received an extra two-day (16 h) structured DMTP delivered by transdisciplinary collaborations through multiple teaching strategies (lectures, simulations, problem-solving lessons, demonstrations, tabletop exercises, discussions, group presentations, and reflections). Readiness for disaster response, consisting of four subscales (emergency response, clinical management, self-protection, and personal preparation), was assessed at baseline and 12 weeks after the intervention. Generalized estimating equations were used as the primary method of data analyses to evaluate the intervention effects.

**Results:**

Ninety-four nurses (94%) completed the study, and 100 nurses were included in the intention-to-treat analysis. While participants in the EG had increased readiness for disaster response after training and at the 12-week follow-up, those in the CG exhibited no differences between baseline and 12-week follow-up. When the group × time interaction was examined, the EG had a greater increase in readiness for disaster response and its four domains, including emergency response, clinical management, self-protection, and personal preparedness after 12 weeks, than the CG.

**Conclusion:**

A two-day structured DMTP utilizing multiple teaching strategies through transdisciplinary collaborations is recommended to enhance hospital nurses' readiness for disaster response. *Implications for Nursing Management*. Nursing leaders should consider incorporating such a structured DMTP into ongoing nursing training as a critical component of professional development programs, thereby strengthening nurses' disaster readiness in hospital settings.

## 1. Introduction

Disasters have engendered significant repercussions characterized by extensive damage, destruction, and loss of life, property, or livelihoods in communities and societies [1]. Worldwide, nations face potential threats from a wide array of disasters, including natural disasters such as earthquakes, hurricanes, floods, landslides, and wildfires, and anthropogenic catastrophes such as terrorist attacks and warfare. These disasters have profound impacts on both individuals and nations, leading to severe consequences [2, 3].

Taiwan, situated in a geographically vulnerable region, faces a wide spectrum of natural disasters and associated hazards. Among these, earthquakes, typhoons, landslides, floods, and industrial accidents have historically affected island nations. Over the past few decades, earthquakes have been one of the most devastating events in Taiwan. Previous disaster epidemiology studies related to the 1999 Chi-Chi earthquake in Taiwan, which claimed 2,415 lives and caused injuries to 11,305 people, revealed that the demand for medical care peaked 12 hours after the earthquake and remained elevated for up to three days [4]. These findings highlight the critical significance of timely disaster response for healthcare in Taiwan.

Disasters can result in a range of adverse outcomes, including infrastructure damage, economic consequences, physical injuries, psychosocial impacts, environmental ramifications, and deprivation of essential services such as water, sanitation, and healthcare, forcing people to leave their homes and communities temporarily or permanently and even lose their lives [3, 5]. Therefore, efforts to decrease the unexpected consequences of disasters and develop strategies to mitigate their impacts remain an imperative research focus. However, to minimize the impact of a disaster and ensure timely and effective responses, a robust frontline medical workforce, particularly nurses, is crucial in disaster areas and local hospitals near the affected sites [6, 7]. Frontline nurses must efficiently assess the severity of patients' injuries and prioritize care based on the level of urgency during a disaster when there may be a high volume of patients in need of care. In addition, nurses are responsible for stabilizing patients experiencing a medical emergency with initial treatment, transferring patients to hospitals or medical facilities, offering coordinated care to patients with other healthcare professionals, and providing emotional and psychological support to patients and their families during disasters [8].

Considering the continuous occurrence of disasters and the negative impacts they entail, nurses' readiness to respond is crucial for mitigating the adverse consequences on affected individuals. Recently, the COVID-19 pandemic has highlighted the necessity of having a national nursing workforce prepared with the knowledge, skills, and abilities to respond [9]. Disaster management is the effort to reduce unexpected consequences and disaster risks [10]. Therefore, to ensure effective and efficient disaster management, it is crucial to enhance frontline nurses' readiness for disaster response, thereby saving lives and mitigating adverse health impacts on affected populations [10, 11].

According to the World Health Organization, most disaster-related deaths occur in healthcare facilities that are unprepared for emergency situations [12]. Adequate preparation and disaster readiness of healthcare personnel, especially nurses who often serve as first responders, can significantly reduce fatalities [8, 13]. Moreover, building resilient health systems through better-prepared medical staff is fundamental [8, 11]. The readiness of nurses not only impacts the immediate effectiveness of disaster response but also significantly affects the overall resilience of health systems and predicts the nurses' willingness to engage in these situations [7] and their capacity to deliver adequate care within a disaster environment [14]. Comprising the largest segment of the healthcare workforce, nurses play a pivotal role in shaping community health outcomes during disasters [15] and managing emergency or disaster medical situations, thereby forming the backbone of the health system. Therefore, it is crucial that nurses possess the requisite knowledge, skills, and competencies, especially readiness, to respond effectively to disasters [16].

However, a significant concern is that many hospital nurses report a lack of readiness for disaster response [15, 17]. Disaster readiness varies significantly across countries, influenced by factors such as economic status, governance, infrastructure, and historical disaster experience [18–21]. For instance, high-income countries like Finland exhibit a proactive approach by incorporating a disaster readiness mindset into governmental strategies [21]. This approach involves identifying potential disaster risks and implementing measures to mitigate them effectively, ensuring a well-coordinated response when disasters occur [21]. In contrast, many countries face challenges in disaster preparedness due to limited resources and infrastructure. Previous studies have highlighted that nurses, especially undergraduate nursing students in these regions, often lack sufficient training and education in disaster management, which is crucial for effective emergency response and clinical management [18, 20, 22]. This gap is evident in regions such as Southeast Asia and parts of Africa, where the frequency of natural disasters like floods and cyclones is high, yet preparedness levels remain inadequate [20, 22]. In Taiwan, a cross-sectional study involving 311 registered nurses at a military hospital found that the majority of nurses demonstrated poor readiness and lacked adequate preparation for disaster situations [11]. Studies have also highlighted that nurses and nursing students with prior disaster-related training and experience in disaster response demonstrate higher readiness and competencies [11, 18]. In India, nurses display moderate levels of preparedness, influenced by various factors such as education, prior disaster experience, mental health, and especially disaster self-efficacy, a vital factor that determines an individual's behavior and performance during disaster situations [23]. In Japan, nurses' disaster readiness has been significantly shaped by the country's frequent exposure to natural disasters. A study examining the disaster preparedness of nurses following the Great East Japan Earthquake found that while they demonstrated moderate levels of overall preparedness, there were notable deficiencies in handling specialized scenarios, such as chemical or biological incidents, and in their psychological preparedness [14]. Thus, the availability and quality of emergency and disaster management training for health professionals vary widely, with many countries offering little to no formal training [14]. Therefore, it is essential to develop structured and effective educational programs to enhance hospital nurses' readiness for disaster response [17].

Disasters also have significant adverse effects on the mental well-being of frontline nurses, who are a high-risk and vulnerable group of medical responders to adverse psychological outcomes [13]. In the context of surging demand, providing high-quality care during disasters is a daunting task. Such situations detrimentally affect the health of healthcare professionals, especially their psychological well-being, owing to the repetitive exposure to traumatic events inherent in their work. Adapting preventive interventions and mitigation strategies targeted specifically at high-risk hospital nurses would be beneficial in decreasing negative outcomes [13]. The lack of adequate training for healthcare providers is a risk factor for adverse psychological health outcomes after disasters [24]. Therefore, designing and incorporating a well-structured disaster management training program into continuous clinical nursing education can mitigate the negative impacts on the mental well-being of hospital nurses who experience disaster events.

Curricula related to disaster training using multidisciplinary methods of simulation and human factor training have been proposed for implementation by organizations such as the Association of American Medical Colleges in the USA, the Government of the Federal Republic of Germany, and the Research Centre in Emergency and Disaster Medicine and Computer Science Applied to Medical Practice in Italy [17]. However, it is currently recognized that there is brief or nonexistent exposure to disaster training within current clinical training curricula worldwide, which may leave hospital nurses unprepared for an intimidating and unfamiliar setting if assisted in the healthcare workforce. Given that various nursing programs have provided education and training in disaster management in recent decades [17, 25–27], most research has focused on nursing students, and most frontline nurses have also reported insufficient readiness or preparedness in responding effectively to disasters [15, 28]. According to a recent study conducted in Greece that evaluated the effects of disaster education on hospital nurses' knowledge, skills, and expertise through evidence-based interventions, the educational intervention resulted in improved knowledge and self-confidence levels among nurses but did not lead to changes in their behavioral intentions [27]. In addition, most existing disaster programs focus on triage skills during disaster response instead of addressing the full spectrum of disaster management, including preparedness, clinical management, emergency response, and self-protection [11, 17]. Tzeng et al. delineated four critical domains of nurses' readiness for disaster response: emergency response, self-protection, clinical management, and personal preparation [11]. These domains align with the International Council of Nurses (ICN) Core Competencies in Disaster Nursing, outlined in 2019, which emphasizes the necessity for nurses to possess both theoretical and practical understanding of disaster preparedness to respond effectively to emergencies [29]. Emergency response entails managing large-scale emergencies through triage, initial stabilization of patients, incident management, medical evacuation, and transportation [8, 11, 19]. Self-protection focuses on the use of protective clothing; handling chemical, biological, radiological, and nuclear decontamination; infection control; and ensuring safety of both patients and nurses [8, 11, 17, 19]. Clinical management involves performing physical assessments, operating equipment in austere environments, and providing specific medical care such as trauma care, management of burn or explosion injuries, first aid, and handling toxic substance injuries [8, 11, 19]. Personal preparation includes acquiring basic survival skills; making physical, psychological, and social plans (law/ethics) prior to engaging in disaster response; communication; recovery; and fostering confidence in collaboration with multidisciplinary teams or peer care [8, 11, 19].

Designing educational programs to enhance frontline nurses' disaster readiness without considering the above key domains of disaster management may not only result in the depletion of manpower and material resources but also fail to achieve the expected outcomes. Given that the ICN's core competencies in disaster nursing and the disaster readiness for response to emergencies and disasters are diverse and extensive, enhancing these competencies and readiness inevitably relies on a variety of teaching strategies. In addition, improving learning effectiveness requires the development of skills across different learning dimensions [30]. Therefore, designing disaster curricula or training programs may require multidisciplinary methods and innovative strategies. However, the development of comprehensive disaster management training programs remains limited, particularly in Taiwan, a region that urgently requires a well-prepared disaster nursing workforce.

This study examined the effectiveness of a two-day structured disaster management training program with multiple teaching strategies to promote hospital nurses' readiness for disaster response. We assume that providing such a well-organized disaster management training program delivered by transdisciplinary collaboration through multiple teaching strategies may enhance nurses' readiness for disaster response, possibly increasing their willingness to respond to disasters and mitigating the negative impacts on hospital nurses' mental well-being in the face of future disaster events. Therefore, the practical purpose of this study was to achieve two outcomes: (1) to improve nurses' readiness for disaster response, enabling them to provide timely and efficient care during disasters and (2) to strengthen nurses' resilience, helping them cope with the trauma associated with disaster response, and empowering them with the skills to take on key roles in disaster management teams.

## 2. Materials and Methods

### 2.1. Study Design

A two-parallel, randomized controlled trial was conducted at a medical centre in northern Taiwan. Eligible hospital nurses were randomly assigned to either a control group (CG), which received regular continuous nursing education, or an experimental group (EG), which participated in an additional two-day (16-hour) structured disaster management training program (DMTP). The readiness for disaster response including four domains (emergency response, clinical management, self-protection, and personal preparedness) was assessed as outcome measures.

### 2.2. Participants

Initially, potential participants from a military medical centre in northern Taiwan, affiliated with the national government and responsible for dispatching personnel to disaster-affected areas in Taiwan or overseas, were recruited by a research assistant. Inclusion criteria were (1) hospital nurses with at least three months' work experience at the local medical centre; (2) aged 20–65 years; (3) full-time employees; (4) able to speak and understand Mandarin; and (5) agreed to participate in the research and to be randomized to one of the two groups. The exclusion criteria were (1) trainee nurses without nursing licenses; (2) new nurses lacking signed contracts because of their inability to work independently; (3) individuals not providing direct care; and (4) those unable to complete the intervention.

Sample size estimation using G^*∗*^ Power (Germany, version 3.1.9) software was based on previous studies with a medium-to-small effect size of 0.15 for the outcome of the expected increase in readiness for disaster response [19, 31, 32], an alpha set at 0.05, number of measurements 2, and a power of 0.80, and each group required 45 hospital nurses [33]. Fifty estimated participants from each group were recruited to account for a 10% loss to follow-up.

### 2.3. Study Cohorts and Interventions

Eligible hospital nurses were randomly assigned to the EG or CG using sealed opaque envelopes following computer-generated random serial numbers by the project investigator. All recruited participants underwent monthly continuous nursing education sessions (30–60 minutes/session) in their workplace units/wards. The participants in the EG received an extra two-day structured DMTP through multiple teaching strategies, such as lectures, simulations, problem-solving lessons, demonstrations, tabletop exercises, discussions, and reflections.

Before implementing the DMTP, we assembled a team of 17 transdisciplinary professionals, including a wilderness survival expert with over 10 years of experience, three nursing specialists in disaster nursing education with more than 10 years of experience, an emergency physician with over 20 years of experience, and 12 clinical practice experts. Among the 12 clinical practice experts, six had experience as nurses supporting disaster events, while the other six were nurse leaders with expertise in emergency/critical care, involving the project investigator/corresponding author. This team held consensus meetings to discuss program content and determine effective teaching strategies for a total of 16 hours, with 2 hours/session weekly for eight weeks. The DMTP, implemented following the consensus protocol developed by the consensus/research team, was structured based on four major domains/themes—emergency response, clinical management, self-protection, and personal preparation [11]—to ensure the validity and veracity of the delivered DMTP.

The outline of the two-day (16-hour) DMTP, as presented in Table 1, included a 2-hour lecture and a 14-hour workshop. The 2-hour lecture covered the concepts of disaster nursing, special concepts of disaster, effects of disasters on health, disaster management and its stages, assessment of possible hazards and vulnerabilities, and stages of planning in disasters. The 14-hour workshop consisted of (a) emergency response: mass casualty and triage, medical evacuation, and delivery; (b) clinical management: trauma care, burn/explosion injury management, first aid treatment, and toxic substance injury management; (c) self-protection: nuclear and biochemical pollution disposal, protective equipment—wear and remove skills—and infection control; and (d) personal preparation: water survival training and rappelling training. In addition, disaster situational responses involve determining the magnitude of a disaster event, planning disaster response, evaluating the health needs of the affected groups, establishing priorities, identifying actual and potential public health problems, determining the resources needed to respond to the needs identified, and collaborating with other professional disciplines and governmental and nongovernmental agencies. Additionally, communication skills were incorporated into the workshop to achieve goals such that the participants could better understand the challenges and complexities of responding to disasters through engagement in hands-on training exercises, role-playing scenarios, and simulations [8, 11, 17].

### 2.4. Measures

Sociodemographic characteristics and readiness for disaster response were collected by a separate research nurse who was blinded to the group assignments. Data on sociodemographic characteristics (age, sex, marital status, and educational level), length of nursing work, position (military or civilian nurse), nursing leader (yes or no), work unit/specialty (critical care units/emergency, general/medical-surgical ward, or others such as outpatient department and operation room), previous disaster training (yes or no), and previous disaster nursing experience (yes, no, or not yet but on list) were collected using self-administered questionnaires. Participants, including head and assistant head nurses of hospitals responsible for overseeing nursing staff, coordinating patient care, and managing resources, were categorized as nursing leaders. The EG was invited to assess their readiness for disaster response at the end of the training program. Both the EG and CG completed the evaluation of readiness for disaster response at 12 weeks. Prior to the study, the tool was reviewed by a panel of experts in disaster management, including five disaster-related experts (content validity index: 0.91), to ensure content validity. A pilot test was also conducted with 10 nurses (2 males and 8 females) to assess the tool's clarity, relevance, and ease of use. Furthermore, quality checks were conducted regularly to ensure the integrity and reliability of the data throughout the study period.

### 2.5. Readiness for Disaster Response

Nurses' readiness for disaster response was assessed using a 40-item self-administered scale with well-established reliability and validity (acceptable convergent validity with 0.84–0.97), including four domains: emergency response (6 items), clinical management (7 items), self-protection (11 items), and personal preparedness (16 items) [11]. The internal consistency reliabilities of the entire questionnaire and its four subscales (emergency response, clinical management, self-protection, and personal preparedness) were 0.96, 0.86, 0.85, 0.88, and 0.97, respectively. Each item was scored from 1 (strongly disagree or very low readiness), 2 (disagree or low readiness), 3 (neutral or average readiness), 4 (agree or high readiness), to 5 (strongly agree or very high readiness) on a five-point Likert scale, with scores ranging from 40 to 200. Each subscale score ranged from 6 to 30, 7 to 35, 11 to 55, and 16 to 80, in emergency response, clinical management, self-protection, and personal preparedness, respectively. Higher scores represented greater readiness for disaster response. The percentage of total scores was categorized into readiness levels: a score percentage above 75% indicated “highly competent or very good,” 50% to 75% indicated “moderately competent or good,” 25% to 50% indicated “low competent or fair,” and below 25% indicated “incompetent or poor.” In addition, the Cronbach's alpha for the scale and its four subscales (personal preparedness, self-protection, emergency response, and clinical management) in this study were 0.92, 0.94, 0.90, 0.82, and 0.76, respectively.

### 2.6. Ethical Consideration

Approval from the institutional review board was obtained (Reference number: 2-103-05-018) from a local medical centre in Taiwan. Participation in the study was entirely voluntary, and the participants could withdraw from the study at any time.

### 2.7. Data Analysis

The study employed SPSS version 16.0 (SPSS Corp., Armonk, NY, USA) for all statistical analyses. Descriptive statistics including means with standard deviations (SDs) and numbers with percentages (%) were utilized to present the characteristics of the study participants. The last observation-carried-forward method of data imputation was used for the intent-to-treat analysis. Baseline characteristic comparisons between groups were conducted using the independent *t*-test or chi-square test. Differences between the groups, both pre- and postintervention, as well as the mean difference between pre- and postintervention within the two groups, were compared using independent *t*-tests. To assess the intervention effects over time, the generalized estimating equations (GEEs) analysis for longitudinal data was applied, considering the significant interaction of group and time (group × time) [34]. All statistical analyses were two-tailed, and significance was set at *p* < 0.05.

## 3. Results

### 3.1. Baseline Characteristics of Participants

Initially, 399 hospital nurses were screened. Of these, 264 nurses declined to participate, and 35 nurses did not meet the inclusion criteria (six had not yet signed contracts with the hospital, four were trainee nurses without nursing licenses, two did not provide direct care, and 23 reported not being able to complete the study intervention). The remaining 100 participants who met the inclusion criteria were randomly assigned: 50 (50%) to the EG and 50 (50%) to the CG.

**Figure 1:**
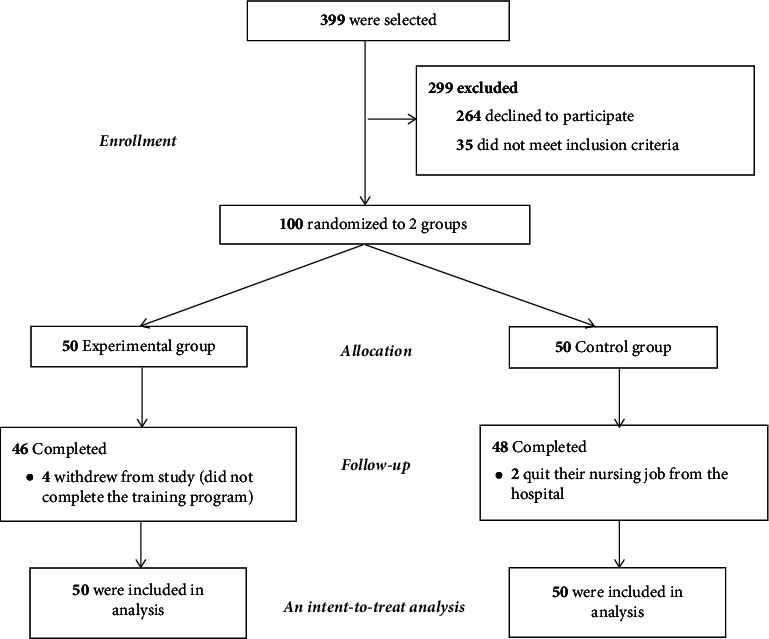
CONSORT diagram of participants' flow through the trial.

Of the 100 eligible hospital nurses, 94 (94%) completed the study. The reasons for missed visits, including withdrawal from the study due to absence on the second day of the training program (*n* = 4) and loss in follow-up due to leaving the nursing job at the hospital (*n* = 2) at the 12-week assessment, are presented in the flow diagram (Figure 1). However, 100 participants were included in the data analysis.

The sociodemographic characteristics, working units, experience of previously received disaster training, and personal participation experience in disaster events of the EG and CG are presented in Table 2. The baseline scores of readiness for disaster response and its four subscales (emergency response, clinical management, self-protection, and personal preparedness) for the two groups are shown in Table 3.

### 3.2. Outcome Evaluation

Table 3 presents the descriptive and univariate analyses of the outcome evaluations. Nurses in the EG had increased readiness for disaster response and its three subscales—emergency response, self-protection, and personal preparedness—immediately after the program and at the 12-week follow-up. However, there were no significant changes in the readiness or its four subscales in the CG. The EG had a remarkably greater increase in the mean differences between pre- and postintervention in readiness for disaster response and its four subscales than the CG.

The effectiveness of the two-day DMTP on readiness for disaster response and its four subscales, including emergency response, clinical management, self-protection, and personal preparedness, assessed through GEE analyses, is shown in Table 4. The analysis revealed a significant interaction between group and time (group × time) regarding readiness for disaster response, indicating that the EG experienced a greater increase at 12 weeks than the CG (*β* = 27.3, *p* < 0.001). Specifically, the EG demonstrated significant improvements in emergency response (*β* = 3.7, *p*=0.002), clinical management (*β* = 3.7, *p*=0.012), self-protection (*β* = 8.4, *p* < 0.001), and preparedness (*β* = 10.5, *p* = 0.003), compared to the CG.

## 4. Discussion

### 4.1. Summary of Findings

Our study demonstrates the effectiveness of a two-day, structured disaster management training program provided by transdisciplinary professionals via multiple teaching strategies in improving hospital nurses' readiness for disaster response, including four domains: emergency response, clinical management, self-protection, and personal preparedness. The program significantly enhanced hospital nurses' disaster management readiness. These findings contribute substantively to the existing literature by confirming that active participation in a structured and comprehensive disaster management training initiative can substantially bolster hospital nurses' preparedness, increasing their confidence and willingness to engage in effective disaster response [17, 35]. Moreover, our results align with previous research, emphasizing the importance of a well-structured training program delivered through collaborative efforts across disciplines and employing varied teaching strategies, ultimately improving response readiness in complex disaster scenarios [17].

The effectiveness of this disaster management training program offers compelling evidence for an optimal educational approach geared towards improving nurses' readiness for disaster response, encompassing emergency response, clinical management, self-protection, and personal preparedness. In light of the steady rise in the availability of disaster training programs over the past two decades, our hypothesis was that creating a structured disaster management training program, delivered by transdisciplinary professionals utilizing multiple teaching strategies, would enhance individuals' knowledge and skills. Furthermore, as previously reported, we believe that it can also enhance their motivation to overcome perceived barriers and indecisive thinking and, most importantly, strengthen their willingness to actively participate in complex disaster situations [17, 35]. The primary focus of the program was to promote readiness for disaster response through a transdisciplinary professional-delivered approach. In addition, the utilization of diverse teaching strategies in the program served as a valuable model for evidence-based nursing education in clinical settings, specifically for hospital nurses.

### 4.2. Comparison with Previous Research

Our study findings highlight that transdisciplinary collaboration brings together experts from various professional fields, such as emergency management, public health, education sciences, and nursing, and ensures a holistic understanding of disasters and their impacts. This collaborative approach allows the design of a comprehensive educational training program for disaster response [17, 36]. Diverse experts foster innovative thinking and problem solving by providing various perspectives, ideas, and solutions, leading to more effective and well-rounded disaster preparedness, response, and management plans [17]. For example, during a consensus meeting before the training program, the invited experts shared diverse perspectives on the program's design. Transdisciplinary professionals collectively identified the vulnerabilities and risks associated with various types of disasters, aiding the development of targeted mitigation strategies and risk reduction measures [17]. In addition, our transdisciplinary teams analyzed resource requirements across multiple sectors and identified efficient allocation strategies, optimizing the use of limited resources and prioritizing critical needs during disasters to simulate real-world scenarios. A recent report stated that the emerging trends in disaster research over the past 20 years, marked by increased international cooperation and the transdisciplinary nature of disaster science, have gained popularity [36]. This demonstrates that valuable lessons can be learned from catastrophes and that these emerging trends serve as a scientific foundation for a clearer understanding of progress in disaster science, providing a reference for rapidly identifying frontier issues in disaster science.

Adopting various teaching approaches such as competency-based, all-hazard, and interprofessional approaches, flipped classrooms, simulations, tabletop exercises, virtual reality, and telenursing care in simulated conditions is the current trend in the design of disaster training programs [17, 37]. Therefore, our study employed multiple teaching strategies, including lectures, simulations, problem-solving lessons, demonstrations, tabletop exercises, discussions, group presentations, and reflections, through transdisciplinary collaborations in designing the disaster management training program for hospital nurses. This program aimed to enhance learning outcomes because individuals have different learning styles and preferences. In addition, participants accommodated various learning styles and engaged in training more effectively, increasing their chances of understanding and retaining information and enhancing learning outcomes. Different teaching strategies promoted active participation and engagement among the participants. Instead of passive learning, participants were actively involved in the learning process through discussions, group activities, and practical exercises. This active engagement facilitates better comprehension, critical thinking, and knowledge application, making training more effective and impactful. In addition, given the rapidly changing nature of disasters, the effectiveness of disaster response remains uncertain, particularly due to the challenges in ensuring the timely arrival of professional rescuers. Therefore, telehealth will play an important role in disaster management. A recent study demonstrated that the quality of telenursing care under simulated conditions was satisfactory during the response phase to disasters at Kerman [37]. Therefore, implementation of telenursing care would be helpful in future disasters; however, more evidence is recommended to support the use of telenursing care training under simulated conditions as an alternative teaching strategy.

Disaster management training requires the development of practical skills such as emergency response procedures, risk assessment, communication protocols, and coordination techniques. Therefore, incorporating hands-on exercises, simulations, and real-life case studies provides opportunities for participants to apply their knowledge and practical skills to make decisions in realistic scenarios. This practical approach helps bridge the gap between theory and practice, building competence and confidence in hospital nurses, thus ensuring their readiness for disaster response. Therefore, using multiple teaching strategies in a disaster management training program not only enhances learning outcomes but also promotes active participation and engagement, facilitates varied perspectives, develops practical skills, improves knowledge retention, fosters collaboration, encourages adaptability, and provides a holistic understanding of the field [17, 36]. These benefits contribute to the overall effectiveness of the training program and equip hospital nurses with effective disaster management strategies.

A recent systematic review reported that, among 23 studies, the majority assessed knowledge (78.3%), attitude (60.9%), or skills (43.5%) following disaster training [35]. This highlights the need for further research on the assessment of readiness for disaster response after such training. Most of the reported disaster programs focused on triage skills during disaster response instead of addressing the full spectrum of disaster management [11, 17]. In addition, the length of disaster training programs ranged from 1 to 28 days, with a median duration of two days [35]. Therefore, we developed and evaluated the effectiveness of a two-day structured DMTP covering the full spectrum of disaster management, including emergency response, clinical management, self-protection, and personal preparedness. Overall, our study found that the DMTP improved hospital nurses' readiness for disaster response, which could be attributed to the enhancement of participants' attitudes, knowledge, and skills in disaster response [35]. In addition, most studies investigating the effectiveness of disaster training programs have used pre- and posttest measures [17]. Therefore, the strength of our study lies in the fact that we conducted a randomized controlled trial to prove the effectiveness of DMTP.

Medical responders are at a high risk of experiencing a wide range of negative psychological health conditions following a disaster. Notably, depression and posttraumatic stress disorder are the most commonly diagnosed conditions among medical responders. A recent report documented that the prevalence of posttraumatic stress disorder among medical workers involved in the earthquake response was 16.4%, highlighting that medical workers involved in responding during disasters should undergo screening for mental health disorders before and after disasters and receive the necessary training regarding stress management and psychological resilience [38]. Particularly, nurses have higher levels of adverse outcomes than physicians and other medical professionals [13]. In addition, when organizations are exposed to disasters, staff members are often unprepared for the potential psychological impacts that can negatively affect their well-being. Fortunately, predisaster training can improve employees' confidence in their ability to cope with disasters [28, 39] and contribute to improving their psychological health [40]. Furthermore, combining reinforcement of emotional intelligence with predisaster training can also facilitate learning outcomes, since emotional intelligence has a significant positive relationship with various components of learning strategies, namely, self-efficacy, rehearsal, critical thinking, cognitive self-regulation, time and study environment management, peer learning, and help seeking [40]. Therefore, lack of structured and comprehensive training is an important risk factor for negative psychological outcomes across all types of disasters [13].

Personal preparedness and self-protection are critical for disaster response management. In challenging disaster environments, emergency services may not be readily available or may become overwhelmed. Individuals who are self-prepared and have basic self-reliance skills are more likely to survive, recover quickly, and be in a position to help others, reducing the risks of injury or death. Therefore, preparedness for self-rescue or self-protection is one of the most important elements of organized and timely emergency response to disaster events [41]. Personal preparedness and self-protection also enable swift responses in the immediate aftermath of a disaster, which is crucial for survival. Additionally, having individuals well-prepared for self-protection during a disaster can alleviate the burden on emergency services, allowing first responders to prioritize those in greatest need [11]. Therefore, personal preparedness and self-protection not only increase individuals' chances of survival and recovery but also contribute to an effective disaster response.

Readiness for disaster response, an important component of disaster management, encompasses the capacity to manage disaster impact quickly and efficiently. Therefore, responding quickly and appropriately to disasters is crucial and depends on frontline nurses' preparedness. Nurses' readiness for disaster response and competencies can vary depending on factors such as their specialty or work area of practice, individual interests, prior experience in in-service training programs, and exposure to deployments in disaster sites [18]. For example, nurses working in emergency departments or critical care settings may have more training and experience in disaster response than those working in other areas of healthcare [28]. However, nurses' readiness for disaster response includes not only clinical management and emergency response but also personal preparedness and self-protection ability [11]. During a disaster event, nurses not only provide first aid and advanced clinical care, monitor physical and mental health needs, allocate resources, conduct efficient communication, and provide crisis leadership [17] but also oversee the use of personal protective equipment, maintain personal emergency supply, prioritize their own safety, utilize appropriate personal protective equipment, and practice infection control measures to minimize exposure to hazards. In addition, according to our previous findings, nurses with prior disaster training were associated with greater readiness for disaster response, including its four major domains: emergency response, clinical management, self-protection, and personal preparedness, after adjusting for potential covariates such as sociodemographics (marital status and educational level), length of nursing work, nursing position, nursing leaders, work unit/specialty, and previous disaster nursing experience [28]. Therefore, the current study aimed to develop a two-day structured disaster management training program that addresses the full spectrum of disaster management, including preparedness, clinical management, emergency response, and self-protection. Fortunately, we discovered the effectiveness of the training program in increasing hospital nurses' readiness for disaster response, both immediately after the program and at the 12-week follow-up.

### 4.3. Limitations and Strengths

There are some limitations in this study that should be considered. First, the geographic region in which this study was conducted may limit its generalizability to other cultural groups. Therefore, the current research findings must be interpreted with caution; more rigorous sampling strategies from multiple sites or hospitals are recommended. Second, although the 12-week follow-up effects after intervention are important, evaluation of long-term follow-up might be necessary. While acknowledging these limitations, it is crucial to recognize the strengths of our study as well. These strengths include the random allocation design, provision of a well-structured disaster management training program with multiple teaching strategies, and a high rate of study completion by the participants (94%).

### 4.4. Contributions and Future Directions

Notably, our study is the first trial to test the effectiveness of a two-day (16-hour) structured disaster management training program that includes four major domains/themes (emergency response, clinical management, self-protection, and personal preparation) through multiple teaching strategies (i.e., lectures, simulations, problem-solving lessons, demonstrations, tabletop exercises, discussions, group presentations, and reflections by transdisciplinary collaborations on readiness for disaster response among hospital nurses). Future studies could apply this training program to confirm its effectiveness across different cultures and focus on investigating competencies, combining knowledge, attitude, skills, readiness, and self-efficacy. This might help to elucidate how the training program improves readiness for disaster response in this population. Moreover, further more studies can (1) conduct longitudinal studies to provide insights into the long-term retention of disaster response skills and identify necessary intervals for refresher training; (2) integrate modern technologies like virtual simulation to potentially elevate training efficacy; (3) explore whether the program not only increases readiness for disaster response but also empowers hospital nurses; and (4) employ focus groups or qualitative methods to examine hospital nurses' perceptions regarding the most helpful aspects of the training. In addition, the feasibility of implementing the intervention in hospitals, educational institutions, and schools is an important next step.

### 4.5. Implications for Nursing Management

In light of our study findings, which reveal that structured disaster management training programs (DMTPs) utilizing multiple teaching strategies and transdisciplinary collaborations significantly enhance nurses' readiness for emergency and disaster response, nursing leaders or managers should actively integrate such DMTPs into ongoing professional development curricula to fortify hospital response readiness and capabilities. Specifically, nursing leaders are encouraged to prioritize these training modules, ensuring they are recurrent and updated regularly to reflect the latest in disaster response protocols and technology. Moreover, it is recommended that these programs be tailored to the specific needs and challenges of the nursing staff within individual healthcare facilities, considering factors such as existing skill levels and potential disaster risks pertinent to geographical locations. Nursing management could also benefit from establishing a feedback loop where participants in these programs contribute insights and suggestions for improvements. This approach fosters a culture of continuous learning and adaptation, thereby improving individual nurse preparedness and enhancing the overall institutional resilience to disasters.

## 5. Conclusions

A two-day structured disaster management training program, delivered by transdisciplinary professionals using multiple teaching strategies, can serve as an effective approach to improve hospital nurses' readiness for disaster response. Such disaster training programs might include innovative methods such as virtual reality and be integrated into ongoing nursing education and future curricula to strengthen disaster readiness across healthcare settings. In addition, continuous educational efforts and periodic refresher training are recommended to maintain and update these critical skills. Nursing leaders or managers should consider incorporating such a structured disaster management training program as a critical component of professional development programs, thereby strengthening nurses' disaster readiness in hospital settings, fostering a culture of continuous learning and adaptation, and enhancing overall institutional resilience to disasters.

## Figures and Tables

**Table 1:** The disaster management training program content in the experimental group.

Content	Teaching strategy	Domain	Time
(1) Concept of disaster nursing, special concepts of disaster, and effects of disaster on health	Lecture	—	2 hr
(2) Disaster management and its stages			
(3) Assessment of possible hazards and vulnerabilities			
(4) Stages of planning in disasters			

(1) Mass casualty and triage	1st-day workshop, six concurrent sessions: lectures, simulations, and problem-solving lessons	*a*, *b*	3 hr. (30 min/each topic or session)
(2) First aid treatment, bone injury, and wound care			
(3) Medical evacuation and delivery/transport			
(4) Childbirth care at disaster sites			
(5) Burn/explosion injury management and fluid infusion			
(6) Management of multiple trauma care			

˙Emphasizing personal safety and protection measures for responders and affected individuals during disasters	Lectures and demonstrations	*c*, *d*	4 hr. (2 hr./each topic or session)
(1) Water survival training			
(2) Rappelling training			

(1) Detection/triage and treatment of nuclear, biological, and chemical contamination/pollution	2nd-day workshop: lectures, simulations, problem-solving lessons, demonstrations, discussions, and reflections	*a*, *b*, *c*	3 hr. (30 min/each session)
(2) Decontamination practice and treatment for nuclear, biological, and chemical substances			
(3) Protective equipment—wear and remove skills			
(4) Infection control in the disaster environment			
(5) Toxic substance injury management			
(6) Management of nuclear, biological, and chemical pollution disposal			

˙Scenario simulation exercises: Conducting simulated emergency scenarios to provide hands-on experience in managing crises, determining the magnitude of the disaster event, planning disaster response, evaluating health needs of the affected groups, establishing priorities, identifying actual and potential public health problems, determining resources needed to respond to the needs identified, and collaborating with other professional disciplines, governmental and nongovernmental agencies, and communication skills	Tabletop exercises, simulations, problem-solving skills, group presentations, group discussions, and debriefing	*a*, *b*, *c*, *d*	3 hr
˙Comprehensive discussions	Group discussions and reflections	—	1 hr

*Note*. ^a^Emergency response, ^b^clinical management, ^c^self-protection, and ^d^personal preparation.

**Table 2:** Comparisons of baseline characteristics between the experimental and control groups.

Variables	Experimental group (*n* = 50)		Control group (*n* = 50)		*t*/*x*^2^	*p*
	Mean ± SD	*n* (%)	Mean ± SD	*n* (%)		
Age (year)	35.5 ± 8.6		34.8 ± 8.1		−0.43	0.67
Length of nursing work (year)	12.6 ± 8.5		11.2 ± 8.5		−0.82	0.42
Gender					0.00	1.0
Female (*n* = 96)		48 (50.0)		48 (50.0)		
Male (*n* = 4)		2 (50.0)		2 (50.0)		
Marital status					3.28	0.35
Married (*n* = 45)		24 (53.3)		21 (46.7)		
Single (*n* = 55)		26 (47.3)		29 (52.7)		
Educational level					3.84	0.14
Associate (*n* = 19)		6 (31.6)		13 (68.4)		
Bachelors (*n* = 53)		27 (50.9)		26 (49.1)		
Masters and above (*n* = 28)		17 (60.7)		11 (39.3)		
Position					1.50	0.31
Military nurse (*n* = 40)		23 (57.5)		17 (42.5)		
Civilian nurse (*n* = 60)		27 (45.0)		33 (55.0)		
Nursing leader					3.66	0.09
Yes (*n* = 33)		21 (63.6)		12 (36.4)		
No (*n* = 67)		29 (43.3)		38 (56.7)		
Work unit/specialty					3.42	0.18
Critical care units/emergency (*n* = 39)		24 (61.5)		15 (38.5)		
General/medical-surgical ward (*n* = 37)		16 (43.2)		21 (56.8)		
Other (*n* = 24)		10 (41.7)		14 (58.3)		
Previously received disaster training					0.41	0.67
Yes (*n* = 33)		18 (54.5)		15 (45.5)		
No (*n* = 67)		32 (47.8)		35 (52.20		
Previous disaster nursing experience					2.11	0.35
Yes (*n* = 8)		5 (62.5)		3 (37.5)		
Not yet (on list) (*n* = 23)		14 (60.9)		9 (39.1)		
No (*n* = 68)		31 (45.6)		37 (54.4)		

*Note.* The data are presented as mean ± SD or number and percentages (%). *P* values were from chi-square test or Student *t-*test, as appropriate.

**Table 3:** Comparison of readiness for disaster response between groups at baseline and 12 weeks.

	Baseline				12 weeks				M.D. between pre- and post-test			
	EG	CG	*t*	*p*	EG	CG	*t*	*p*	EG	CG	*t*	*p*
	(*n* = 50)	(*n* = 50)			(*n* = 50)	(*n* = 50)			(*n* = 50)	(*n* = 50)		
	Mean (SD)	Mean (SD)			Mean (SD)	Mean (SD)			Mean (SD)	Mean (SD)		
Readiness for disaster response	111.8 (27.9)	121.2 (25.7)	−1.48	0.14	139.8 (23.3)	122.7 (27.8)	3.34	0.001	28.5 (28.4)	1.4 (14.6)	5.94	<0.001
Emergency response	16.3 (4.9)	17.4 (3.9)	−1.20	0.23	20.1 (3.5)	17.5 (4.5)	3.23	0.002	3.8 (4.6)	0.18 (3.1)	4.67	<0.001
Clinical management	23.0 (6.1)	24.6 (5.4)	−1.41	0.16	26.6 (4.4)	24.8 (5.1)	1.91	0.06	3.8 (5.5)	0.18 (3.8)	3.87	<0.001
Self-protection	26.7 (7.9)	29.2 (7.0)	−1.68	0.10	36.2 (6.9)	30.3 (8.6)	3.78	<0.001	9.5 (8.8)	1.1 (5.3)	5.73	<0.001
Personal preparedness	47.0 (13.9)	50.1 (13.1)	−1.16	0.25	56.9 (11.2)	50.1 (13.1)	2.79	0.01	10.4 (13.9)	−0.02 (3.8)	4.91	<0.001

*Note.* M.D., mean difference between pre- and post-test (12 weeks); *P* values were from independent *t-*test.

**Table 4:** Evaluation of the effectiveness of nurses' readiness for disaster response based on GEE analysis.

	EG (*n* = 50)			CG (*n* = 50)			Between group		Interaction: group (EG) × time^c^				
	Mean ± SD	*β*	Within	Mean ± SD	*β*	Within	*β*	*p* ^b^	*β*	SE	95% C.I.		*p*
			*p* ^a^			*p* ^a^					Lower	Upper	
Readiness for disaster response									27.3	7.4	12.8	41.7	<0.001
Baseline	111.8 ± 27.9	—	Ref	121.2 ± 25.7	—	Ref	−10.2	0.06					
After training	145.8 ± 28.1	34.7	<0.001	—	—	—	—	—					
12 weeks	139.8 ± 23.3	28.7	<0.001	122.7 ± 27.8	1.4	0.79	17.1	0.001					
Emergency response									3.7	1.2	1.3	6.0	0.002
Baseline	16.3 ± 4.9	—	Ref	17.4 ± 3.9	—	Ref	−1.1	0.23					
After training	21.0 ± 4.4	4.7	<0.001	—	—	—	—	—					
12 weeks	20.1 ± 3.5	3.8	<0.001	17.5 ± 4.5	0.2	0.83	2.6	0.001					
Clinical management									3.7	1.5	0.8	6.5	0.012
Baseline	22.7 ± 5.8	—	Ref	24.6 ± 5.4	—	Ref	−1.8	0.10					
After training	27.1 ± 5.4	4.3	<0.001	—	—	—	—	—					
12 weeks	26.6 ± 4.4	3.8	<0.001	24.8 ± 5.1	0.2	0.86	1.8	0.05					
Self-protection									8.4	2.1	4.2	12.6	<0.001
Baseline	26.7 ± 7.9	—	Ref	29.2 ± 7.0	—	Ref	−2.5	0.09					
After training	38.9 ± 7.8	12.2	<0.001	—	—	—	—	—					
12 weeks	56.9 ± 11.2	9.5	<0.001	30.3 ± 8.6	1.1	0.48	5.9	<0.001					
Personal preparedness								—	10.5	3.6	3.45	17.5	0.003
Baseline	46.4 ± 13.6	—	Ref	50.1 ± 13.1	—	Ref	−3.7	0.17					
After training	58.9 ± 12.3	12.4	<0.001	—	—	—	—	—					
12 weeks	56.9 ± 11.2	10.4	<0.001	50.1 ± 13.1	−0.02	0.99	6.8	0.01					

*Note.* EG: experimental group; CG: control group; C.I., confidence interval; *P* values were from GEE models, with a group × time interaction term characterizing the intervention effect of interest; ^a^reference group: baseline; ^b^reference group: control group; ^c^reference group: group (CG) × time.

## Data Availability

Data are available from the corresponding author upon reasonable request.
